# Supplementary material for: Proteome and Glycoproteome Analyses Reveal the Protein N-Linked Glycosylation Specificity of STT3A and STT3B
Source: Cells. 2022 Sep 6;11(18):2775. doi: 10.3390/cells11182775 (PMC9496733; doi:10.3390/cells11182775)
Supplement: Supplementary file 1 [file cells-11-02775-s001.zip › Supplementary Figures.pdf]

Supplementary Figures

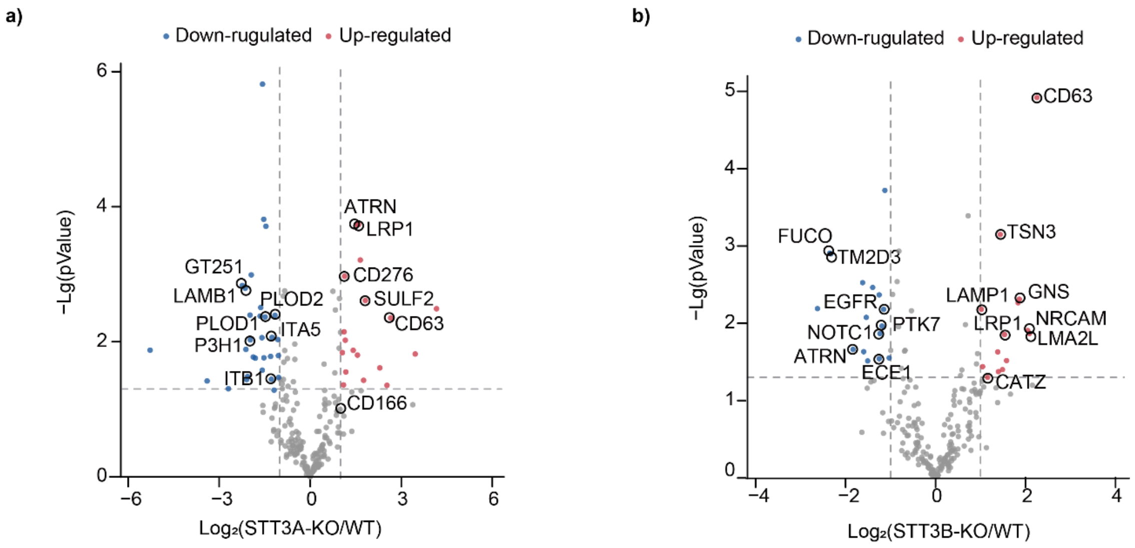

Figure S1. Volcano plot of glycoproteins. a) Expression changes of glycoproteins in STT3A-KO. b) Expression changes of glycoproteins in STT3B-KO.

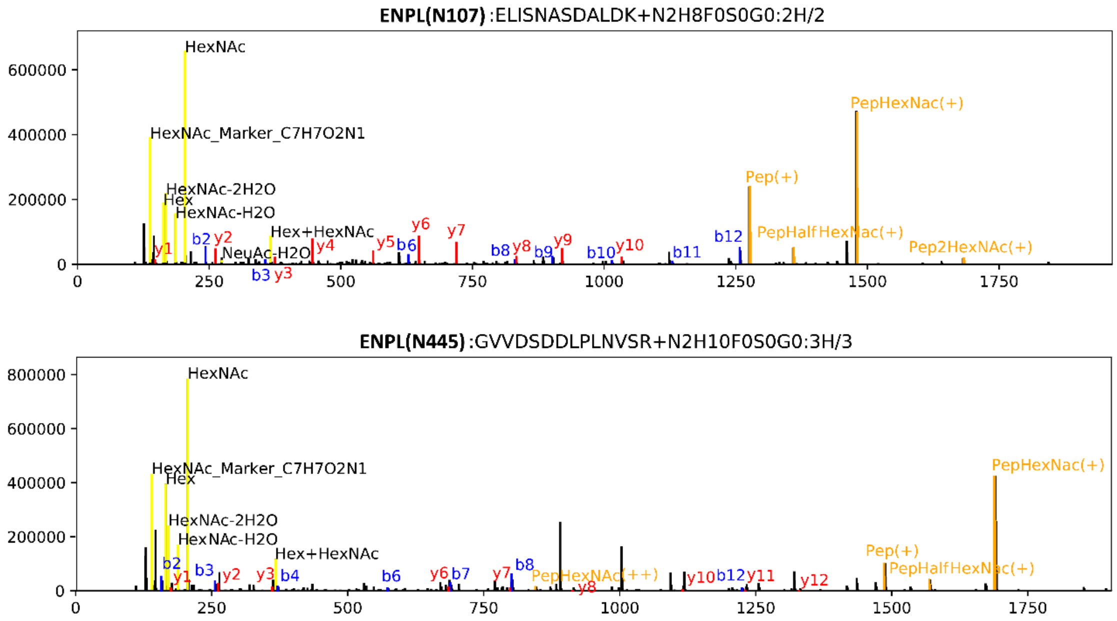

Figure S2. Annotated mass spectrum of hyperglycosylated glycopeptides of ENPL.
